# Development of a Web Portal for Physical Activity and Symptom Tracking in Oncology Patients: Protocol for a Prospective Cohort Study

**DOI:** 10.2196/resprot.9586

**Published:** 2018-05-15

**Authors:** Michael Marthick, Haryana M Dhillon, Jennifer A Alison, Birinder S Cheema, Tim Shaw

**Affiliations:** ^1^ Chris O'Brien Lifehouse Camperdown Australia; ^2^ Research in Implementation Science and eHealth Group Faculty of Health Sciences University of Sydney Sydney Australia; ^3^ Centre for Medical Psychology & Evidence-Based Decision-Making School of Psychology University of Sydney Sydney Australia; ^4^ Discipline of Physiotherapy Faculty of Health Sciences University of Sydney Sydney Australia; ^5^ School of Science and Health Western Sydney University Penrith Australia

**Keywords:** physical activity, patient portals, fitness trackers, neoplasms

## Abstract

**Background:**

Significant benefits accrue from increasing physical activity levels in people with a history of cancer. Physical activity levels can be increased using behavioral change interventions in this population. Access to Web portals and provision of activity monitors to provide feedback may support behavior change by encouraging patient engagement in physical therapy. The Web portal evaluated in this study will provide a system to monitor physical activity and sleep, for use by both clinician and patient, along with symptom and health-related quality of life tracking capabilities.

**Objective:**

The aim of this study was to outline a protocol for a feasibility study focused on a Web-based portal that provides activity monitoring and personalized messaging to increase physical activity in people with cancer.

**Methods:**

Using a longitudinal cohort design, people with cancer will be serially allocated to 3 intervention cohorts of 20 participants each and followed for 10 weeks. Cohort 1 will be provided a wearable activity monitor and access to a Web-based portal. Cohort 2 will receive the same content as Cohort 1 and in addition will receive a weekly activity summary message. Cohort 3 will receive the same content as Cohorts 1 and 2 and in addition will receive a personalized weekly coaching message. Feasibility of the use of the portal is the primary outcome.

**Results:**

Results are expected in early 2018. Outcome measures will include goal attainment and completion rate.

**Conclusions:**

This study will provide information about the feasibility of investigating eHealth initiatives to promote physical activity in people with cancer.

**Registered Report Identifier:**

RR1-10.2196/9586

## Introduction

### Physical Activity and Cancer

There is consistent evidence that exercise prescribed during or following completion of cancer treatment is safe and feasible and reduces clinically important symptoms and side effects, including fatigue, low mood, and loss of muscular strength and aerobic fitness [1-3]. In addition, evidence is emerging that exercise may positively impact treatment delivery, such as improvements in chemotherapy completion rates, number of dose reductions, as well as decreased cancer recurrence rate and improved overall survival in some cancers [2,3]. Despite this evidence, achieving behavioral change in cancer populations in relation to exercise has been challenging [4]. Physical activity levels typically decline significantly during intensive cancer treatments, such as chemotherapy and radiation therapy, and often do not return to prediagnosis [5] or minimum recommended levels [6].

### eHealth and Web-Based Portals

eHealth can be broadly defined as the integration of devices, communication, and data in health care [7]. The use of eHealth tools, such as Web portals, wearable activity trackers, and personalized text and email messaging, may increase the capacity for delivering individualized, scalable, and cost-effective physical activity–based behavioral change initiatives in this population.

Research involving patients with a history of cancer indicates that most have positive attitudes toward the use of eHealth methods in their management and care of cancer [8]. The use of Web portals by patients with a chronic condition such as cancer is a growing area of eHealth research. Web portals can have many uses including patient access to personal medical records, appointments, medications, communication with health professionals, and decision support tools [9-13]. The use and application of Web portals provide opportunities to improve physical activity behavior change. Patients with access to Web portals tend to have greater engagement in their treatment, lower treatment distress, increased treatment satisfaction, and improved communication with health professionals [9-13].

Web-based methods such as Web portals also provide a new method to record, track, and relay patient-reported outcome (PRO) measures to health professionals involved with patient care. Symptom tracking and reporting using PRO measures have been shown to improve patient outcomes in cancer care [14]. Historically, paper- or phone-based interactive voice response systems have been used [15,16]. The novel approach of using a Web portal for the collection and communication of PRO may provide a more effective platform for health care professionals to facilitate long-term behavior change in cancer survivors.

### Wearable Activity Trackers

Accelerometers (or activity trackers) are small, lightweight devices worn on the wrist or hip. Activity trackers have been used extensively in clinical research of physical activity [17]. Recently, they have become more available to consumers with the release of relatively low-cost and accessible products. Commercially available accelerometers have been validated as an accurate measure of physical activity and sleep metrics [17,18], including models from Misfit, Fitbit, and Garmin. In people with cancer, accelerometers provide a reliable and feasible method to measure, monitor, and encourage physical activity [18]. A major issue when using commercially available activity trackers in clinical practice is that individual patient data are not readily available to health professionals involved in their care. Web portals can provide a mechanism for data transfer through an application programming interface that supports interaction between software components, giving both patients and health care professionals access to real-time data.

### Personalized Messaging

The use of email and SMS (short message service) text messaging is ubiquitous in the general population. The use of SMS text messaging as a behavioral change intervention, including personalized messaging, has emerged as a promising approach to promote positive behavior change [19]. Personalized messages (PMs) are often sent daily or weekly, aiming to improve lifestyle behaviors including improving physical activity levels, sleep quality, and nutrition habits. PMs are likely most effective when tailored to each patient rather than generalized to broader audiences [20,21]. The intended benefit of regular PMs is to elicit long-term and sustainable behavior change.

Our study aims to evaluate the feasibility of a dual-facing (patient and clinician) Web portal, which incorporates synchronized data from activity trackers, symptom management, and PMs as a method of monitoring and improving care for people with cancer who attend an Australian comprehensive cancer care center.

## Methods

### Study Design

The study will be a prospective longitudinal cohort design. Participants will be enrolled serially into 3 successive cohorts: the first 20 participants enrolled were entered into Cohort 1, the second 20 into Cohort 2, and the final 20 into Cohort 3. No form of random allocation will be used.

**Figure 1:**
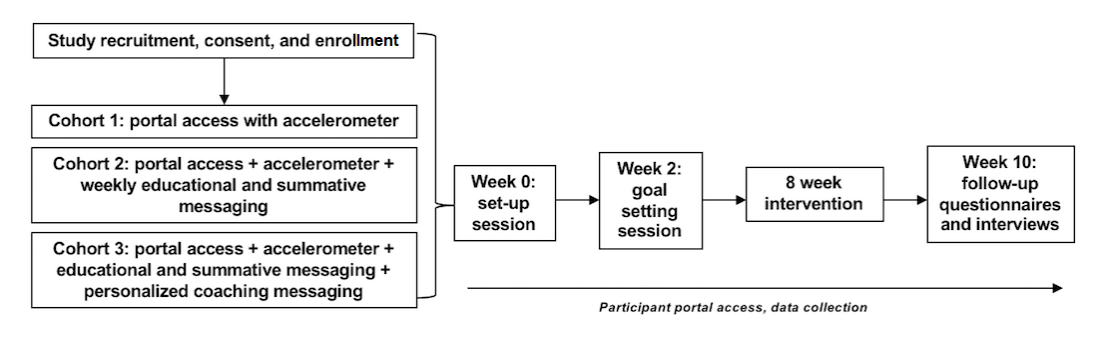
Schematic summary of the study.

Cohort 1 will be provided Web portal access with an accelerometer for 10 weeks. Cohort 2 will be provided Web portal access, accelerometer, and an additional weekly, automated summary message that details average scores over the last week, along with specific educational content such as information on cancer-related fatigue and nutrition. Cohort 3 will receive the content received by Cohort 2 and additional personalized behavioral change messaging from an accredited exercise physiologist (EP). Figure 1 shows the schematic research design.

### Participant Inclusion and Exclusion Criteria

A convenience sample of patients registered with the cancer care center, either currently undergoing treatment or who have completed treatment within the last 6 months, will be invited to participate in the study.

Participant inclusion criteria will be as follows: (1) aged 18 years or older, (2) have an Eastern Cooperative Oncology Group Performance Status score of 0 to 2, and (3) have smartphone and Internet access readily available.

Participants will be excluded if they (1) are unable or have limited ability to speak English and (2) have any condition that would compromise their ability to understand the participant information or give informed consent.

### Development of the Web Portal

The Web portal was developed with an industry partner (Springday Pty Ltd), under a collaborative research agreement.

An initial focus group involving patient consumer representatives (n=2) and clinicians (n=8) working in oncology was organized to obtain feedback on user experience, content, and design. During the focus group, a draft design was discussed, and suggested improvements were documented and incorporated where possible.

**Figure 2:**
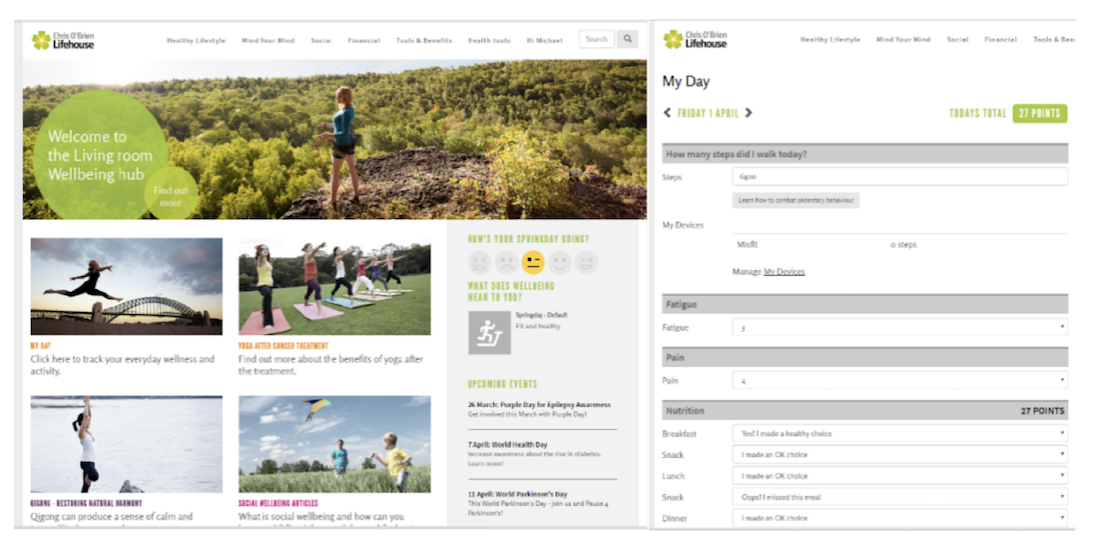
Example of a patient-facing Web portal home page and virtual diary.

There are 2 main components of the Web portal: (1) the participant interface and (2) the clinician interface. The participant interface provides a website for individuals to synchronize data from their personal activity tracker and view their activity and symptom data (Figure 2). It also provides access to a Web-based library containing information about cancer, cancer treatment, and the benefits of adopting healthy lifestyle behaviors, including better nutrition, exercise, and sleep habits. Links to supportive networks and government-supported cancer information websites will be provided via the Web portal.

**Figure 3:**
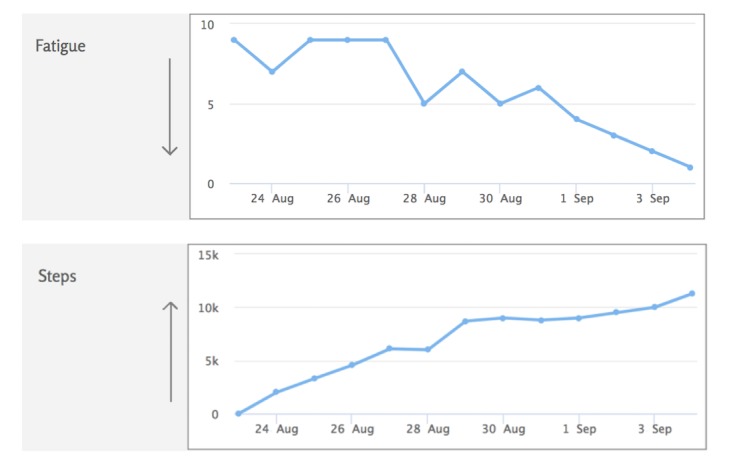
Example of an individual data snapshot in clinician facing "coaching dashboard".

The clinician interface, also called the *coaching dashboard*, provides clinician users with an overview of all participants under their care. It summarizes participant demographics, activity tracker, and symptom trends both individually and overall (Figure 3). PRO measure data are available, with data trends visible. Activity tracker information is securely synchronized from the participant’s smartphone to the Web portal every 30 min using an application programming interface.

### Activity and Sleep Tracker

The study will use the commercially available Misfit Shine activity monitor. Misfit Shine was chosen because of its 3- to 6-month battery life, Bluetooth synchronization capability, and water-resistant characteristics. This device has been validated as a physical activity measurement instrument in healthy populations [22]. Participants enrolled in the study are able to bring their own device if they currently own one from the Misfit, Garmin, or FitBit product ranges as the Web portal has the capacity to synchronize data from these devices.

### Recruitment Procedure

There will be rolling serial recruitment into each of the 3 cohorts. Once 20 participants have been recruited for Cohort 1, recruitment will then start for Cohort 2. After 20 additional participants have been recruited for Cohort 2, recruitment will start for Cohort 3. Eligible patients will be identified by either a clinical nurse consultant, physical therapist, or a supportive care and integrative medicine specialist. The clinician will provide a written participant information sheet to potentially eligible participants. Potential participants consenting to be contacted will be screened to confirm eligibility and will be provided verbal information about the study. Participants will be registered into the study after signing the informed consent form during their individual orientation session. Due to the nature of working with a population who are undergoing intensive cancer treatment, a slight attrition is expected to occur in each cohort.

### Intervention

The intervention will include evidence-based components of education, goal setting, monitoring, feedback, and motivation as described by Michie et al [23]. It is a behavioral change intervention, primarily focused on remote symptom monitoring and increasing physical activity levels. The intervention is divided into 3 major components covered in individual sessions: (1) orientation, (2) goal setting, and (3) individual messaging. All 3 cohorts will complete sessions 1 and 2, and additionally, Cohort 2 will receive automated summary messages, and Cohort 3 will receive individualized coaching messages.

#### Orientation Session

An initial, 30-min, one-to-one session will be conducted in person with the participant and an EP. The EP will orient the participant regarding the use of the activity monitor and the Web portal. The participant will be involved in entering their demographic data into the Web portal, downloading the MisFit Shine mobile app, and synchronizing this device with the Web portal. An information pack including the Misfit Shine user manual and instructions for the Web portal will be given to each participant.

#### Goal Setting Session

Each cohort will have an initial 2 weeks of Web portal access to determine baseline activity levels. Following this initial data collection phase, a 15-min goal planning session will be conducted with an EP either face-to-face or by telephone. This session will aim to set a daily step goal for each participant to achieve over the duration of the study, typically 10% greater than the participant’s median daily value over the last 2 weeks. Step goals will be capped at 15,000 steps per day.

#### Web Portal Access

Participants will have access to the Web portal for 8 weeks after the goal setting session, 10 weeks in total. During this time, data, both from the activity tracker and those which are manually entered, will be collected and will be accessible for both the participant and study investigators. Participants will be asked to wear the activity tracker for the duration of the study and synchronize their device daily. Participants will able to track their fatigue and pain scores daily on the Web portal. These symptoms will be rated on a Likert scale from 0 (none) to 10 (worst imaginable) using a drop-down menu.

#### Personalized Messaging

Following the goal setting session in week 2, the Web portal will provide 2 types of coaching messages to participants in Cohorts 2 and 3 through weekly emails. Cohort 2 will receive a summative message providing participants with a summary of their exercise history, sleep duration, and an overview of their fatigue and pain scores.

Cohort 3 will receive the same general summative message as Cohort 2 as well as weekly health coaching messages that will be tailored based on the participant’s progress over the last week. This will also take into consideration messages received from the participant, which may include information such as barriers, goals, and treatment status.

### Technical Support

Participants will have access to technical support for the duration of the study. This includes telephone and email support with the study coordinator during standard business hours on week days.

### Study Procedures

Table 1 depicts the investigations and timings from preregistration to week 10. At the first face-to-face session, baseline questionnaires will be completed on the Web portal. The following validated PRO measures will be used:

1. Edmonton Symptom Assessment Scale: An 18-item, patient-rated symptom visual analogue scale developed for use in assessing cancer-related symptoms [24].
2. Functional Assessment of Cancer Therapy-General: It is a 27-item compilation of general questions divided into 4 primary health-related quality of life (HRQoL) domains: physical well-being, social/family well-being, emotional well-being, and functional well-being. It is considered appropriate for use for patients with any form of cancer [25].
3. Cancer Behavior Inventory-Brief version: This self-efficacy scale [26] will also be given to the participants to complete.

An additional study-specific feedback questionnaire will be remotely administered via a Web survey to assess the following: (1) participant satisfaction with the device and Web portal, (2) perceived appropriateness of the device and Web portal, (3) ease of use of the Web portal, (4) intent to continue using the device and Web portal, (5) device and Web portal technology issues, (6) personal technology use, (7) telehealth usability, and (8) general feedback.

### Semistructured Interviews

Qualitative, semistructured interviews will be conducted with participants to capture, in depth, their experience of the intervention. This method enables the researchers to explore in-depth insights into the experiences and perspectives of individual participants. Interviews will be conducted either face-to-face or via telephone by an individual not involved in delivering the intervention or the participant’s clinical care. Each interview will be audio-recorded and transcribed verbatim. The transcripts will be coded and themes will be identified using a thematic approach to analysis in a framework structure. Interviews will be conducted with consenting participants in each cohort of the study and will continue until saturation of themes has been achieved.

### Data Management

Paper copies of signed consent forms will be stored securely at the participating site. Data synchronized from the activity monitor or entered manually will be stored on the Web portal Web server. The participant Web portal uses HTTPS to encrypt traffic; database servers employ encryption at rest; and users have individual passwords. During the data analysis phase, participant Web portal data will be extracted and stored on a secure, customized electronic Research Electronic Data Capture (REDCap) database housed on a secure server. REDCap is a browser-based software for capturing clinical and translational research data created by the Vanderbilt University. Access to the server will be restricted to study investigators only and via individual passwords.

### Primary and Secondary Outcomes

The primary outcome of interest is the feasibility of the program. The intervention would be deemed feasible if a compliance rate of >70% is observed.

Compliance is defined based on 2 measures:

1. Log-ins: a patient is defined as compliant if they have more than 2 log-ins over the 10-week study period.
2. Questionnaires: a patient is defined as compliant if they complete the follow-up questionnaire at week 10.

For the study to be deemed feasible, >70% of the participants need to comply with both criteria, not just one.

The secondary objectives of the study are as follows:

1. To describe goal attainment; the number of individuals who are eligible and take up the program; the rate of program completion; satisfaction with the intervention; self-efficacy related to change in lifestyle factors; and clinical changes including symptom and HRQoL scores
2. To analyze Web portal data to determine median daily step count, weekly email engagement (Cohorts 2 and 3), and number of PMs sent (Cohort 3)
3. To compare accelerometer data from the first week (week 1) and final week (week 10).

**Table 1:** Data collection schedule. Checkmarks indicate time points when data were collected.

Data collection	Preregistration	Baseline	Week 2	During the intervention	Week 10
Eligibility check	✓				
Consent		✓			
Demographics		✓			
Goal setting			✓		
Functional Assessment of Cancer Therapy-General		✓			✓
Edmonton Symptom Assessment Scale		✓			✓
Cancer Behavior Inventory-Brief version		✓			✓
Pain score (0-10)			✓	✓	
Fatigue score (0-10)			✓	✓	
Activity tracker data			✓	✓	
Web portal usage data				✓	
Adverse events				✓	
Participant survey					✓
Interviews					✓

### Data Analysis

Baseline demographics will be summarized as frequency (%) for categorical variables and as mean (SD) or median (interquartile range, IQR) for continuous variables, depending on the distribution. The number of compliant participants within each cohort will be summarized as frequency (%). The number (%) of patients who attained their step goal will be summarized weekly and by cohort, along with the median number of weeks where goals were attained.

The daily step count will be summarized at week 1 and week 10 for each cohort as mean (SD) or median (IQR). The mean difference between week 1 and week 10 will be displayed alongside the corresponding 95% CI. Data will be analyzed on the days the accelerometer was worn, defined as more than 250 steps.

Quality-of-life questionnaires will be summarized as median (IQR) or mean (SD) at the initial and week 10 visit for each cohort group. The mean difference between time points will be displayed alongside the 95% CI.

The frequency (proportion) of opened emails will be summarized for Cohort 2 and Cohort 3 by each week of the study, and the number of PMs opened by Cohort 3 will be summarized as frequency (%).

The number of symptoms reported will be used to investigate the association of baseline characteristics with engagement in the study. A Mann-Whitney *U* test will be used to compare the number of symptoms between categorical variables, and Spearman correlation will be used to investigate the association with continuous variables.

### Ethics

Permission to conduct this study has been granted by the Royal Prince Alfred Hospital Human Research and Ethics Committee (X16-0051). All participants will provide written informed consent, and the study will be conducted in accordance with the Declaration of Helsinki and applicable national guidelines.

## Results

The project was funded in 2016, and enrollment was completed at the end of 2017. Data analysis is currently under way, and the first results are expected to be submitted for publication in 2018.

## Discussion

Reduced physical activity levels during cancer treatment can lead to increased symptom burden and consequently reduced quality of life. There is no single solution to facilitate positive behavior change across a population of people with cancer in active treatment; however, the innovative use of technology may benefit a proportion of the population. Patients with cancer who have tertiary education, who are undergoing intensive treatment, and those who report reduced HRQoL express a more positive perception of and openness to use of Web portals [8,27]. Despite an overall positive perception of eHealth interventions, there are varying perceptions of their usability from both patients and clinicians, although benefits to self-management and outcomes have been demonstrated [8,9].

It is acknowledged that this study will recruit a heterogeneous patient sample, with various tumor types and treatment stages represented. However, the decision to include a heterogeneous sample was deemed appropriate for a feasibility study and is also pragmatic for recruiting the required sample within a short time frame as well as the generalizability of the findings. Where patient numbers are sufficient, we will explore the impact of the intervention on specific tumor groups, stages of disease, and point in the cancer treatment trajectory to inform the design of any future efficacy trials.

The utility of Web portals for clinicians and clinician-patient relationships is an important benefit of such systems. This study does not include data review or interactions with medical specialists; rather, it will be limited to an exercise professional. Depending on feasibility, future iterations will include such components.

Determining the feasibility of the intervention is the major objective of this study. The inclusion of qualitative interviews strengthens the study and will help to guide future development of the system and studies. User-designed health systems have been shown to increase functionality, specificity, and uptake [28].

Results from this prospective cohort study will add to the body of evidence surrounding the use of eHealth initiatives to facilitate physical activity behavior change and symptom tracking in people with cancer. It is anticipated that the results of this pilot will inform the design of a future randomized controlled trial that will be adequately powered to assess clinically relevant outcomes.

## Abbreviations

EP: exercise physiologist
HRQoL: health-related quality of life
IQR: interquartile range
PM: personalized message
PRO: patient-reported outcome
REDCap: Research Electronic Data Capture
SMS: short message service
